# Rumen and lower gut microbiomes relationship with feed efficiency and production traits throughout the lactation of Holstein dairy cows

**DOI:** 10.1038/s41598-022-08761-5

**Published:** 2022-03-22

**Authors:** Hugo F. Monteiro, Ziyao Zhou, Marilia S. Gomes, Phillip M. G. Peixoto, Erika C. R. Bonsaglia, Igor F. Canisso, Bart C. Weimer, Fabio S. Lima

**Affiliations:** 1 Department of Population Health and Reproduction, School of Veterinary Medicine, University of California, Davis, CA 95616 USA; 2 Department of Veterinary Clinical Medicine, College of Veterinary Medicine, Sichuan Agricultural University, Chengdu, China; 3 Department of Veterinary Clinical Medicine, University of Illinois Urbana-Champaign, Urbana, IL USA; 4 Department of Animal Sciences, University of Florida, Gainesville, FL USA

**Keywords:** Applied microbiology, Microbial communities, Microbiome, Bacteria

## Abstract

Fermentation of dietary nutrients in ruminants' gastrointestinal (GI) tract is an essential mechanism utilized to meet daily energy requirements. Especially in lactating dairy cows, the GI microbiome plays a pivotal role in the breakdown of indigestible plant polysaccharides and supply most AAs, fatty acids, and gluconeogenic precursors for milk synthesis. Although the contribution of the rumen microbiome to production efficiency in dairy cows has been widely researched over the years, variations throughout the lactation and the lower gut microbiome contribution to these traits remain poorly characterized. Therefore, we investigated throughout lactation the relationship between the rumen and lower gut microbiomes with production efficiency traits in Holstein cows. We found that the microbiome from both locations has temporal stability throughout lactation, yet factors such as feed intake levels played a significant role in shaping microbiome diversity. The composition of the rumen microbiome was dependent on feed intake. In contrast, the lower gut microbiome was less dependent on feed intake and associated with a potentially enhanced ability to digest dietary nutrients. Therefore, milk production traits may be more correlated with microorganisms present in the lower gut than previously expected. The current study's findings advance our understanding of the temporal relationship of the rumen and lower gut microbiomes by enabling a broader overview of the gut microbiome and production efficiency towards more sustainable livestock production.

## Introduction

Ruminants are unique animals capable of harvesting the most from complex nutrients that humans cannot digest^1^. This unique characteristic of ruminants comes from their symbiosis with the GI tract microbiomes (rumen and lower gut microbiomes)^2^. Specifically, in cattle, these two microbiomes ferment dietary nutrients and deliver to the host as much as 70% of their energy and 90% of their daily protein requirements for body maintenance, growth, production, and reproduction^3–5^. Interestingly, dairy cattle vary in their efficiency of obtaining nutrients from the diet. Moderate heritability has partially explained this variability in feed efficiency (h^2^ = 0.36 ± 0.06)^6^. Thus, given the importance of the GI tract fermentation of livestock, several studies have achieved considerable progress in understanding the relationship of the GI tract microbiome with the production efficiency of ruminants^1,7–11^.

In beef cattle and lambs, for instance, microorganisms throughout the GI tract have been associated with feed efficiency, sometimes even being considered as important as those in the rumen^10,11^. For dairy cows, most studies have focused on understanding the rumen microbiome relationship with production efficiency and not on the whole GI tract microbiome as in other species^1,7–11^. The rumen is, in fact, an essential contributor to production efficiency, being the primary fermentation site in ruminants and a pre-absorptive site in the GI tract^12^. However, the lower gut microbiome contributes to almost 10% of the total metabolizable energy intake of dairy cows by further fermenting nutrients that escape degradation and absorption in previous compartments of the GI tract. Thus, considering the daily energy requirements of lactating dairy cows, other compartments of the GI tract may also play an essential role in the production potential of these animals^13^. Despite previous studies reporting the association of rumen microorganisms to production efficiency^14,15^, associations of the lower gut microbiome with the production potential of dairy cows have not been much explored. Although studies demonstrated variability in rumen microbiome between dry and lactating dairy cows^15^, these stages of a dairy cow cycle represent significant differences in diets that often do not occur throughout lactation.

Therefore, we hypothesized that the integration of rumen and lower gut microbiomes throughout lactation would have greater association with feed and milk production efficiency in Holstein dairy cows than the rumen microbiome alone. To test this hypothesis, we characterized the rumen and lower gut microbiomes throughout the lactation of primiparous Holstein cows and their associations with feed and milk production efficiency. Herein, we demonstrated an important relationship of the lower gut microbiome throughout lactation with feed efficiency and the milk production potential of dairy cows.

## Results and discussion

Dairy cows in the study had on average 33,339 ± 9193 sequence reads identified from their rumen and 44,619 ± 11,983 sequence reads from their lower gut microbiomes following the DADA2 denoising pipeline. From these sequence reads, a total of 7825 amplicon sequence variants (ASVs) were identified in the rumen, and 3627 ASVs were identified in the lower gut after taxonomy assignment. Although absolute values of diversity indices may vary based on several factors (e.g. sequencing depth), total ASVs found in both microbiomes showed greater diversity (*P* < 0.001) in the ruminal fluid compared to the feces.

Other studies also reported greater diversity in the rumen than the lower gut microbiome^16,17^, and a similar trend has been reported in the pre-weaning period of Holstein calves^18^. The greater diversity of microorganisms in the rumen may be expected because of the wider variety of substrates available for fermentation in this organ that represent ~ 50% of the GI tract in cattle^19,20^. However, a lower concentration of microorganisms in the rumen may also be expected because of a possible faster passage rate of digesta there than in the lower gut, which may increase the dilution rate of a media considering that consumption tends to be consistent in dairy cows^19,21^. From a lower gut perspective, these findings align with our hypothesis. A lower variety of substrates available for fermentation in the lower gut may lead to a lower diversity of microorganisms growing in the media^19,22,23^. Therefore, more specialist and not generalist microorganisms may thrive under a lower diversity of nutrients^23^. A decrease in genetic variation should be further explored in future studies as this would allow a more direct association of these microorganisms with production traits related to undigested dietary nutrients, such as production efficiency. Furthermore, a potential greater retention of digesta suggest that the lower gut may be an important site contributing to fermentation in the GI tract of ruminants and a significant player of feed and production efficiency in dairy cows.

### Effects of the day of lactation on rumen and lower gut microbiomes

**Figure 1:**
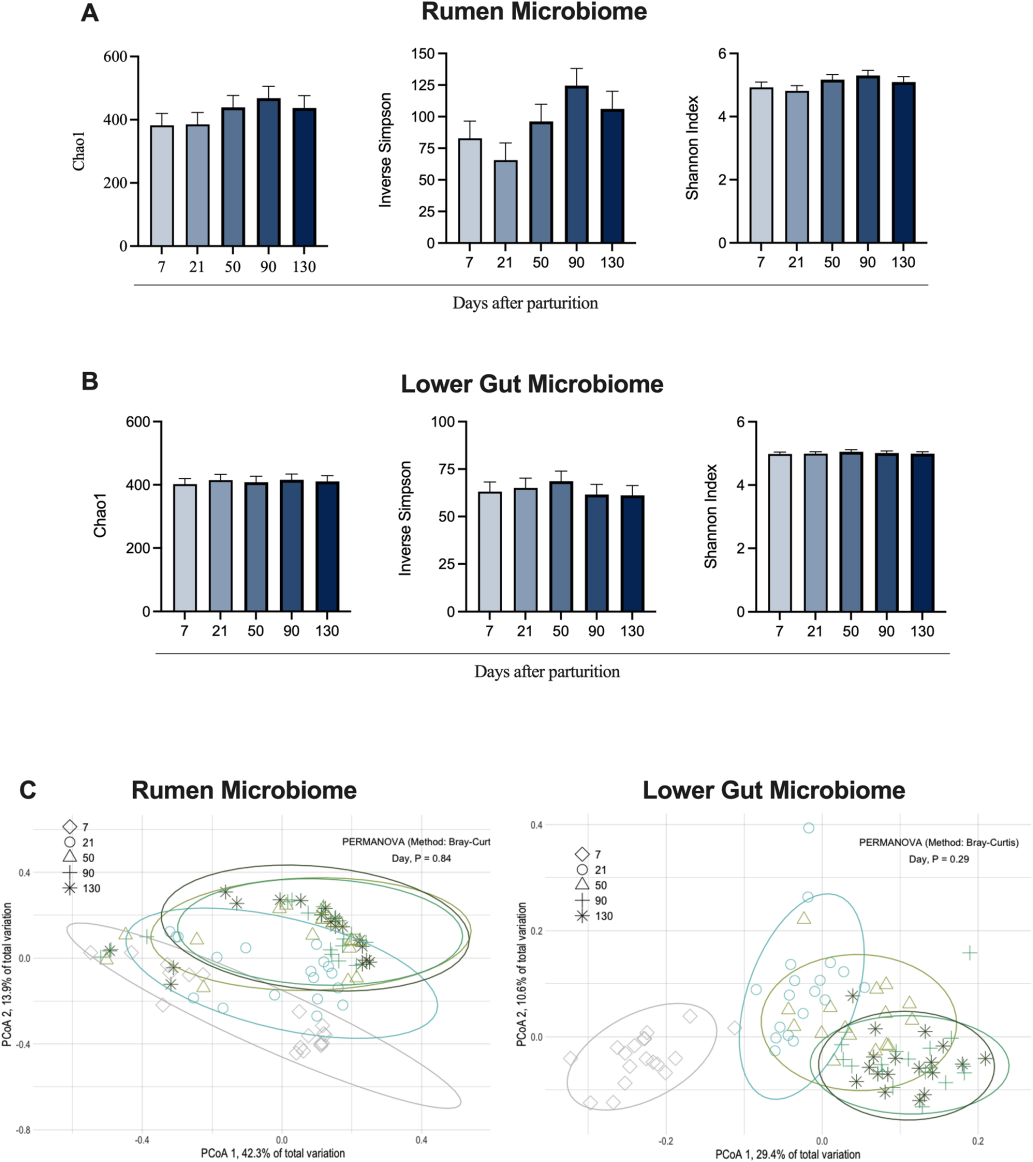
Effects of the day of lactation on alpha- (Chao, Inverse Simpson, and Shannon) and beta-diversity indexes from the rumen and lower gut microbiomes in first lactation Holstein cows. Permutational multivariate analysis of variance (PERMANOVA) was calculated using the Bray–Curtis method and used to test dispersion centroids in the principal coordinate analysis (PCoA). Significance was declared at *P* ≤ 0.05; however, no significant differences were observed for any diversity index.

One of the primary goals of the current study was to investigate the rumen and lower gut microbiomes shifts throughout the main stages of lactation to test our hypothesis. No major effects of lactation day other than a non-statistical apparent difference for day 7 in the lower gut were observed in both microbiomes for alpha and beta-diversity indexes (Table 1 and Fig. 1). There was no association between the day of lactation with any of the variables tested in the rumen and lower gut microbiomes. Of interest, early lactation has been reported to be a period of disturbances in the lower gut microbiome^16^. Specifically, a study evaluating the fecal microbiome differences between days 1 and 14 of lactation recorded changes over time at the phylum and genus level^16^. Using 16S rRNA gene sequencing, the authors reported most of the changes to be likely associated with a decrease bacteria genera (*Bacillus*, *Clostridium_sensu_stricto_1*, *Clostridium_sensu_stricto_6*, *Escherichia-Shigella*, *Klebsiella*, and *Staphylococcus*) that could be a source of pathogenic strains until day 14, suggesting that differences in the lower gut microbiome could be linked to an improvement in overall lower gut health as lactation progressed.

**Table 1:** Permutational multivariate analysis of variance (PERMANOVA) to test associations of production efficiency variables in first lactation Holstein cows.

Item	*P*-Values	
	Rumen	Lower Gut
Day	0.84	0.29
DMI, kg/d	< 0.01	< 0.001
**Milk production, kg/day**		
ECM	< 0.01	< 0.001
Milk fat	< 0.001	< 0.01
Milk lactose	< 0.001	< 0.01
Milk protein	< 0.001	< 0.001
**Feed efficiency**		
Residual feed intake, RFI	0.04	0.04
*RFI variables*		
MBW, kg	< 0.001	< 0.001
BEC, Mcal/d	< 0.001	0.26
NESec, Mcal/d	< 0.01	< 0.001
*RFI variables, unit/kg DMI*		
MBW	0.01	0.19
BEC	0.03	0.01
NESec	< 0.001	< 0.001
**Production efficiency, kg/kg DMI**		
Energy-corrected milk, a.k.a. GFE	0.18	< 0.01
Milk fat	0.12	0.03
Milk lactose	0.30	0.19
Milk protein	0.49	< 0.01

Regarding the rumen microbiome, most structural changes are expected earlier in the ruminant life, especially between birth and weaning^18^. Indeed, it has been reported that microbial and functional changes extensively happen towards the ones found in mature ruminants during the pre-weaning period. However, when it comes to the variation of the rumen microbiome and its functionality in adult ruminants, such as those during a dairy cow's lactation, stability is observed, and a limited day-to-day variation across early, middle and late lactation happens instead^24^. Thus, our results support the hypothesis that the rumen and lower gut microbiomes of first lactation Holstein dairy cows are resistant to changes throughout lactation. Thus, the sampling day should not affect the outcome of interest if the animal is under similar dietary and environmental conditions.

### The association of the rumen and lower gut microbiomes with feed efficiency

**Figure 2:**
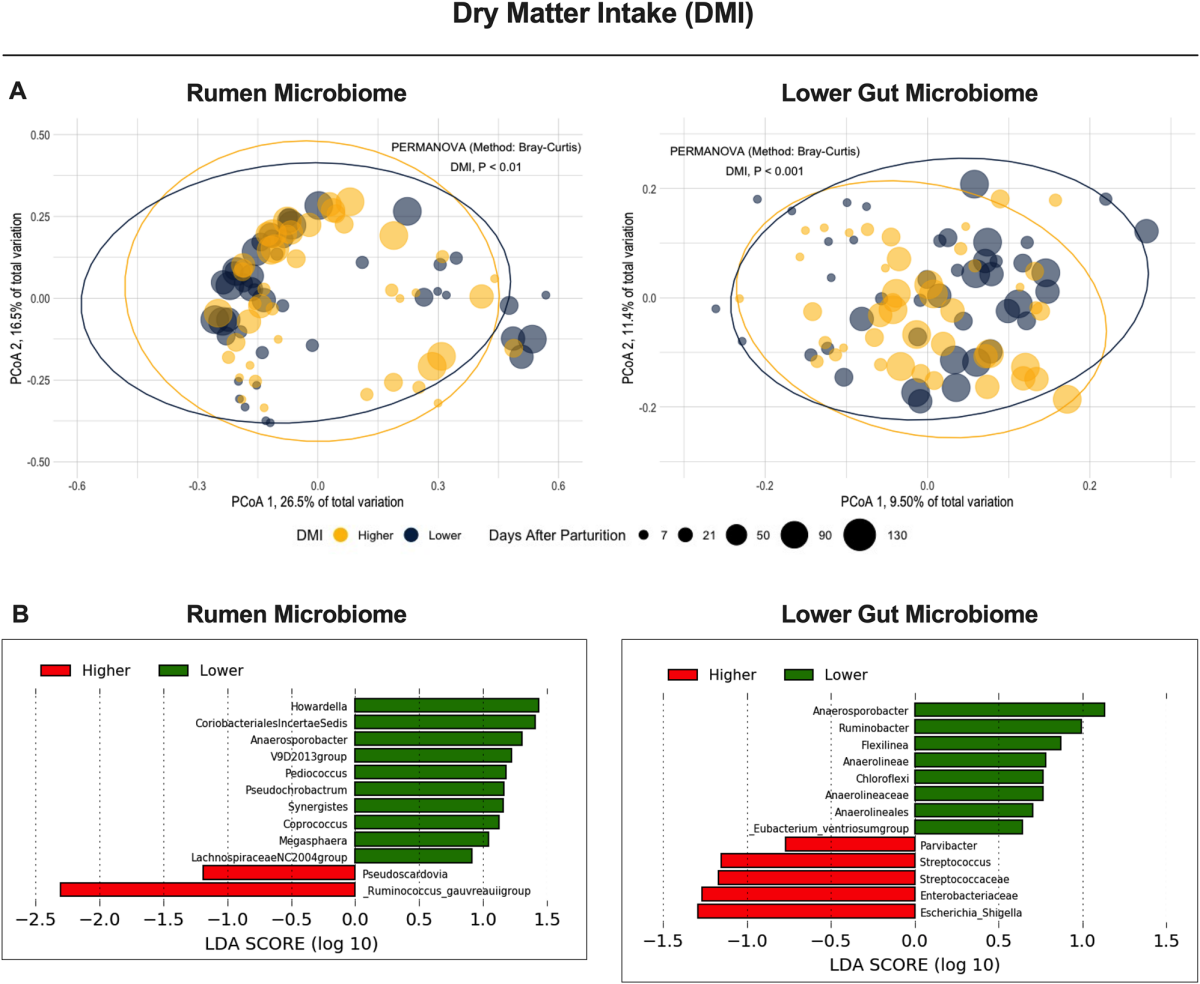
Beta-diversity analyses in the rumen and lower gut microbiomes between first lactation Holstein cows with different levels of dry matter intake (DMI; kg/d). (**A**) Principal Coordinate Analysis (PCoA) and permutational multivariate analysis of variance (PERMANOVA) in the cows grouped by dry matter intake; Significance was declared at *P* ≤ 0.05. (**B**) Linear discriminant analysis of effect size (LEfSe) using Kruskal–Wallis, Wilcoxon, and linear discriminant analyses to detect microorganisms associated with different levels of DMI (kg/d); ASVs significant in all three tests displays a LDA score > 2.0, and the remaining ASVs were significant in at least two other tests.

To test the hypothesis that the integration of the rumen and lower gut microbiomes would have greater association with efficiency variables in dairy cows than the rumen microbiome alone, we investigated associations of the rumen and lower gut microbiomes with the feed consumption of these animals. Here, dry matter intake (DMI kg/d) was used to measure feed consumption by the cows. Cows consuming higher DMI did not significantly differ in the alpha-diversity index (*P* > 0.05) for their rumen and lower gut microbiomes compared to those in the lower-end ones (Supplementary Fig. 1). For beta-diversity, there was a significant association of the level of consumption with the rumen and lower gut microbiomes (Fig. 2; *P* = 0.01 and *P* = 0.001, respectively). Interestingly, rumen fill is one of the major mechanisms regulating feed consumption^25^, suggesting microorganisms in the rumen may also contribute to regulating feed consumption. In our study, cows consuming higher DMI had a greater ruminal relative abundance of *Ruminococcus_gauvreauii_group*, while the greater ruminal relative abundance of *Howardella* was associated with lower DMI levels. *Ruminococcus_gauvreauii_group* is a fibrolytic bacterium^26^ that may contribute to an enhanced fermentation of fiber in the rumen, thus, allowing a faster turnover of ruminal digesta and feed consumption in cattle. On the other hand, *Howardella* is an ureolytic bacteria^27^, suggesting an increase in the recycling of ruminal urea may happen in these cows due to decreased feed consumption. The latter represents a lower protein intake by the cow compared to the other group, which may increase the recycling of urea to the rumen and the growth of microorganisms associated with its degradation. These extreme examples in both cases show how ASVs could perhaps be used as indicators of production traits in ruminants, warranting further research. Furthermore, because DMI level is one of the significant drivers of milk production (kg/d), all the parameters evaluated from here on were based on the correction of the DMI level to avoid the confounding effect of this variable (Table 1; *feed* and *production efficiency*).

**Figure 3:**
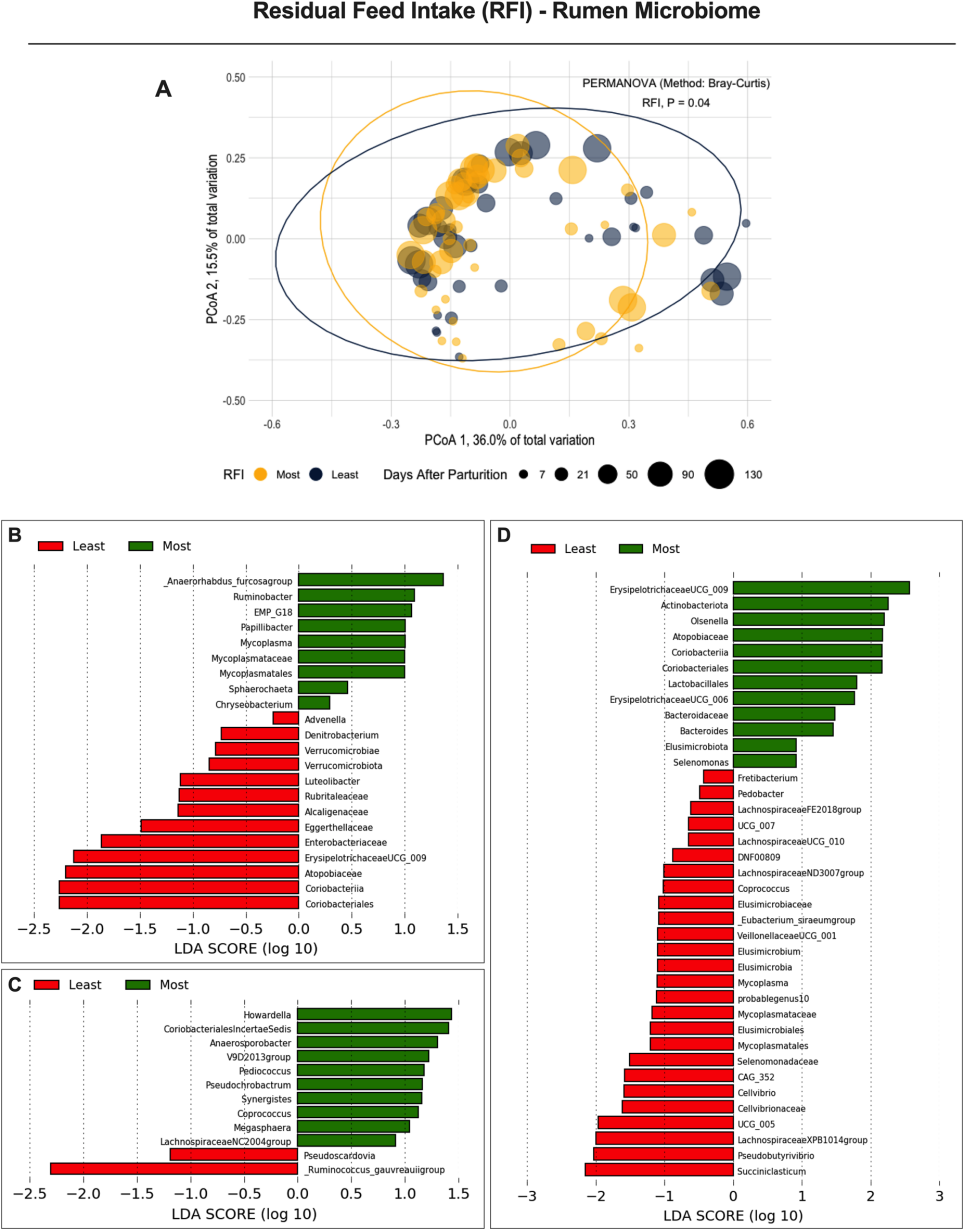
Beta-diversity analyses from the rumen microbiome of first lactation Holstein cows differing in residual feed intake (RFI): (**A**) Principal Coordinate Analysis (PCoA) and permutational multivariate analysis of variance (PERMANOVA) in the cows grouped by RFI; Significance was declared at *P* ≤ 0.05. (**B**–**D**) Linear discriminant analysis of effect size (LEfSe) using Kruskal–Wallis, Wilcoxon, and linear discriminant analyses to detect microorganisms associated with RFI variables corrected for DMI (kg/d) [(**B**) metabolic body weight, MBW; (**C**) body energy changes, BEC; and (**D**) net energy secreted in the milk, NESec]. Amplicon sequence variants significant in all three tests display a LDA score > 2.0, and the remaining ASVs were significant in at least two other tests.

**Figure 4:**
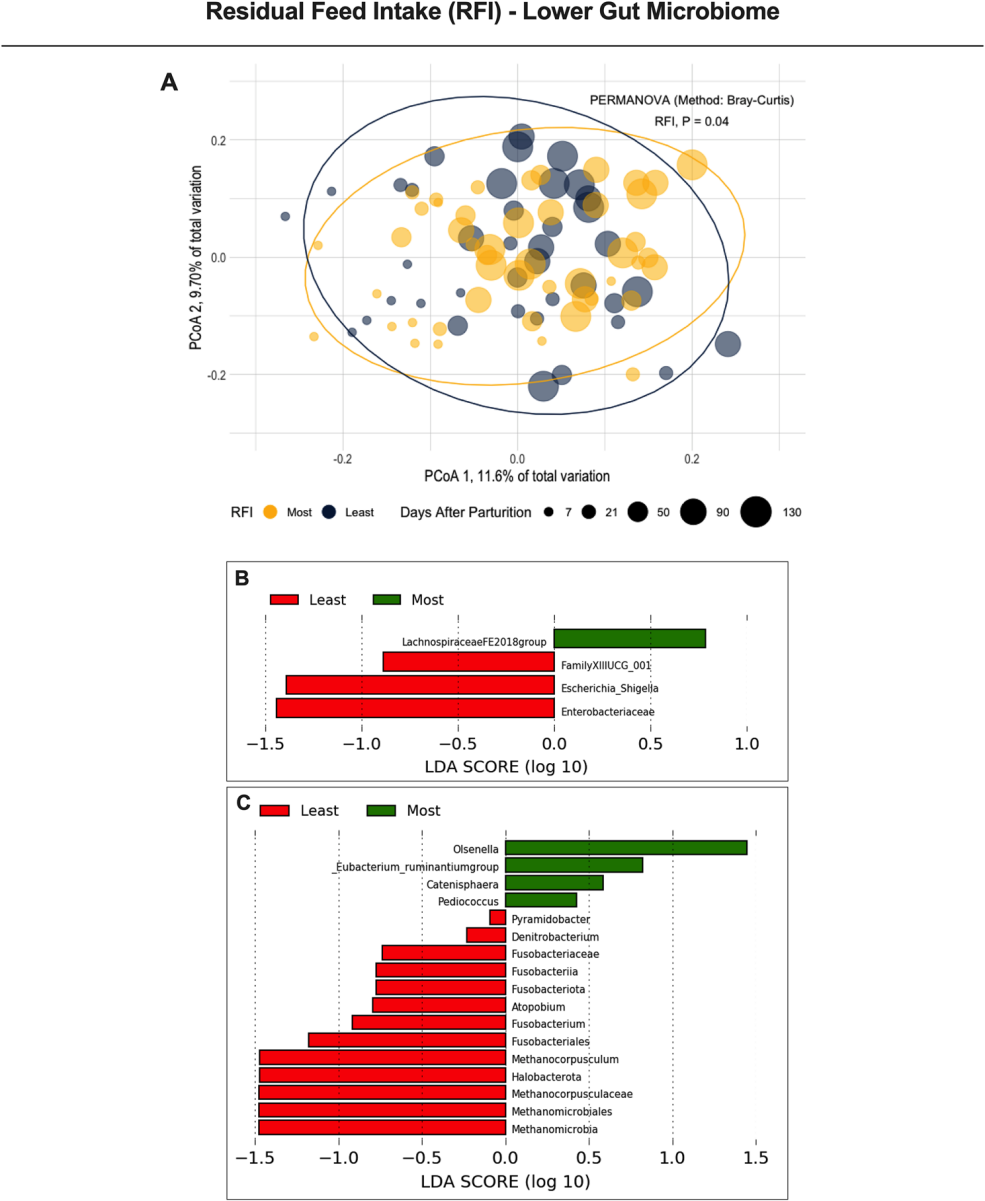
Beta-diversity analyses from the lower gut microbiome of first lactation Holstein cows differing in residual feed intake (RFI). (**A**) Principal Coordinate Analysis (PCoA) and permutational multivariate analysis of variance (PERMANOVA) in the cows grouped by RFI; Significance was declared at *P* ≤ 0.05. (**B**,**C**) Linear discriminant analysis of effect size (LEfSe) using Kruskal–Wallis, Wilcoxon, and linear discriminant analyses to detect microorganisms associated with RFI variables corrected for dry matter intake [(**B**) body energy changes, BEC; and (**C**) net energy secreted in the milk, NESec]. Amplicon sequence variants significant in all three tests display a LDA score > 2.0, and the remaining ASVs were significant in at least two other tests.

The association of residual feed intake (RFI), which is a measure of feed efficiency, with the rumen and lower gut microbiomes was first assessed. The residuals of feed intake were obtained from the difference between the predicted and the observed DMI of the cows throughout the study^28^. Here, an association of rumen and lower gut microbiomes was observed with cows that were classified as the least or most efficient based on their RFI values (Table 1). Such associations are displayed for the rumen and the lower gut in Figs. 3 and 4, respectively. An association of both microbiomes with RFI in beef cows has been reported^10^, and herein we identified a similar relationship in lactating Holstein cows. Therefore, by using the lower gut microbiome instead of the rumen to provide insights on RFI, more non-invasive strategies could emerge to access the production potential of lactating dairy cows. Furthermore, it is well established that the lower gut, cecum, and fecal microbiomes are similar in ruminants, which emphasizes the importance of utilizing these sites as strategies to avoid invasive sampling protocols when evaluating their production efficiency^10^.

Core microbiomes (set of microorganisms characteristic of a phenotype^29^) were characterized based on ASVs associated with RFI or with each of the variables used to calculate RFI [DMI (kg/d), metabolic body weight (MBW kg), NE_L_ secreted in the milk (NESec Mcal/d), and body energy changes (BEC Mcal/d)]. The latter integrated core microbiome was more diverse than the core generated by ranking the cows according to only RFI. Thus, the relationship of the integrated core microbiome for RFI was explored using the rumen and lower gut microbiomes. These RFI variables were also corrected by DMI to avoid the confounding effect from feed consumption. Except for the lack of association of the lower gut microbiome with MBW after the correction for DMI, all other RFI variables (corrected for DMI) had associations with both microbiomes throughout lactation (Figs. 3 and 4). To our knowledge, this is likely the first study reporting these associations. Together, these core microbiomes may better explain differences in the harvesting efficiency of the cows. Understanding the microorganisms associated with greater uptake of nutrients in the GI tract may help improve the predictability of DMI.

### The association of the rumen and lower gut microbiomes with milk production efficiency

The second part of our hypothesis explored the integration of the rumen and lower gut microbiomes to test if both site together would have a greater association with milk production efficiency than the rumen microbiome alone. First, we investigate associations of the rumen and lower gut microbiomes with the output of products [(e.g., energy-corrected milk (ECM kg/d)] by dairy cows; labeled here as production traits. Cows producing higher ECM within the group of animals enrolled in the study did not differ in alpha-diversity indexes for their rumen and lower gut microbiomes (data not shown). However, for beta-diversity, there was an association of production of milk components reported here as milk fat, lactose, and protein yield with both microbiomes (Table 1). Previous studies also found an association of the rumen microbiome with production traits during the lactation of dairy cows^8,15,30^. Specifically, in the study by Lima et al.^15^, the rumen microbiome accurately predicted milk production even when rumen samples were collected in the prepartum period (adjusted R^2^ = 0.82 for first lactation cows). Here, we showed the lower gut microbiome is also associated with these production traits of dairy cows, which may bring advantages over the rumen microbiome due to a less invasive sampling procedure for feces.

**Figure 5:**
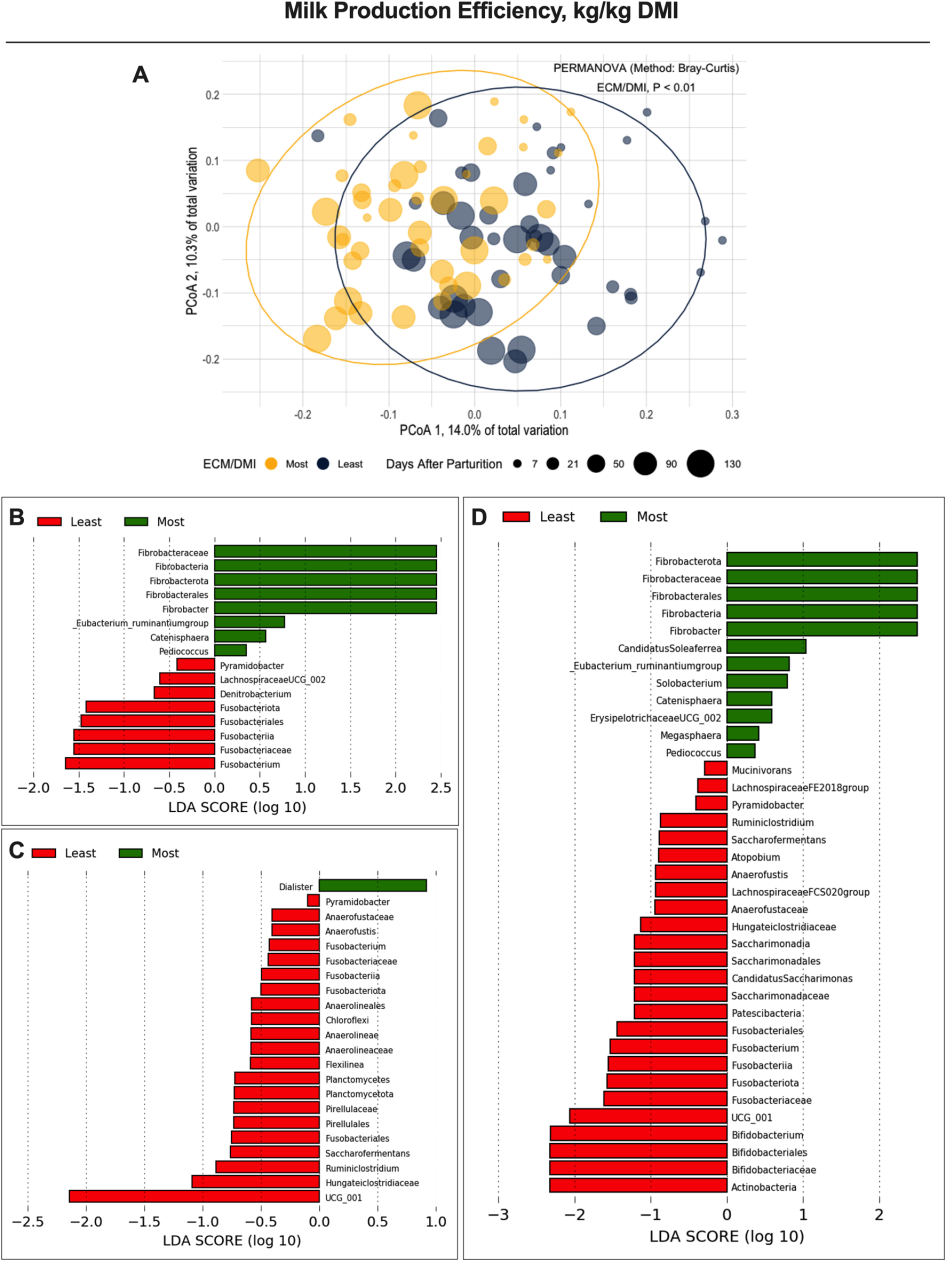
Beta-diversity analyses from the lower gut microbiome of first lactation Holstein cows differing in production efficiency (kg/kg DMI). (**A**) Principal Coordinate Analysis (PCoA) and permutational multivariate analysis of variance (PERMANOVA) in the cows grouped by milk production efficiency; Significance was declared at *P* ≤ 0.05. (**B**–**D**) Linear discriminant analysis of effect size (LEfSe) using Kruskal–Wallis, Wilcoxon, and linear discriminant analyses to detect microorganisms associated with production efficiency variables [(**B**) efficiency of producing energy-corrected milk, ECM; (**C**) efficiency of producing milk fat, MFE; and (**D**) efficiency of producing milk protein, MPE]. Amplicon sequence variants significant in all three tests display a LDA score > 2.0 and the remaining ASVs were significant in at least two other tests.

Furthermore, ECM and milk components (e.g., milk fat kg/d) were also corrected for DMI to evaluate if the rumen and lower gut microbiomes could be associated with the efficiency in producing those outputs. Interestingly, despite previous studies reporting an association of ruminal microorganisms with production efficiency^1,8,30^, the lower gut microbiome in the current study had a better association with production traits than the rumen microbiome (Table 1). Among the production traits measured in this study, the lower gut microbiome of cows grouped based on milk production efficiency was the most distinct of the PCoA and PERMANOVA analyses (Fig. 5). A possible explanation may be related to undigested dietary nutrients reaching the large intestine, which is likely one of the major factors determining the microbial community composition of the GI tract of these animals^31,32^. Cows with lower efficiency in producing ECM, milk fat, and milk protein from the same amount of substrate consumed (i.e., DMI) may have more undigested nutrients reaching the large intestine and likely have more microorganisms associated with these nutrients.

There is also the possibility of cows with higher efficiency in producing milk and its components to have greater efficiency in extracting more out of undigested nutrients reaching the large intestine, which would enhance the overall energy extracted from dietary nutrients. Previous studies focusing on the rumen microbiome show that when Holsteins cows are fed high-forage diets, more microorganisms associated with carbohydrate-active enzymes (CAZymes) are present in the rumen^33^. These enzymes are associated with enhanced cellulose, hemicellulose, and pectin degradation^34,35^, which are likely the major nutrients reaching the lower gut of ruminants. In our study's LEfSe analysis for the lower gut microbiome, which shows ASVs primarily associated with each efficiency group (least vs*.* most efficient; Fig. 4), we observed an increase of fibrolytic bacteria in animals more efficient in producing milk and milk protein. The major difference was the increase in *Fibrobacter* and *Eubacteria Ruminantium*, which, if metabolically displayed CAZymes activity, could confirm such a mechanism of improved efficiency.

Except for milk fat production efficiency that had most ASVs associated with this trait being from the least efficient animals, these findings suggest a possible link between the balance of ASVs associated with the most and least efficient animals, as shown in the LEfSe analysis. Cows with lower efficiency in producing milk, milk fat, and milk protein had a greater overall lower gut relative abundance of *Fusobacterium*, *Ruminococcaceae UCG-001*, and *Actinobacteria*. Interestingly, *Fusobacterium* was negatively associated with all production efficiency traits in the lower gut microbiome. Species of *Fusobacterium*, such as *Fusobacterium necrophorum*, have been reported to present in higher abundance in cattle fed diets rich in grain^36^, which aligns with our findings suggesting that more efficient animals are likely to have more undegraded substrates reaching the lower gut. In other words, least efficient animals are likely to have more highly fermentable carbohydrates reaching the lower gut, possibly due to lower efficiency in processing these nutrients in previous compartments of the GI.

### Correlation of the lower gut microbiome with feed intake and production efficiency in dairy cows

**Figure 6:**
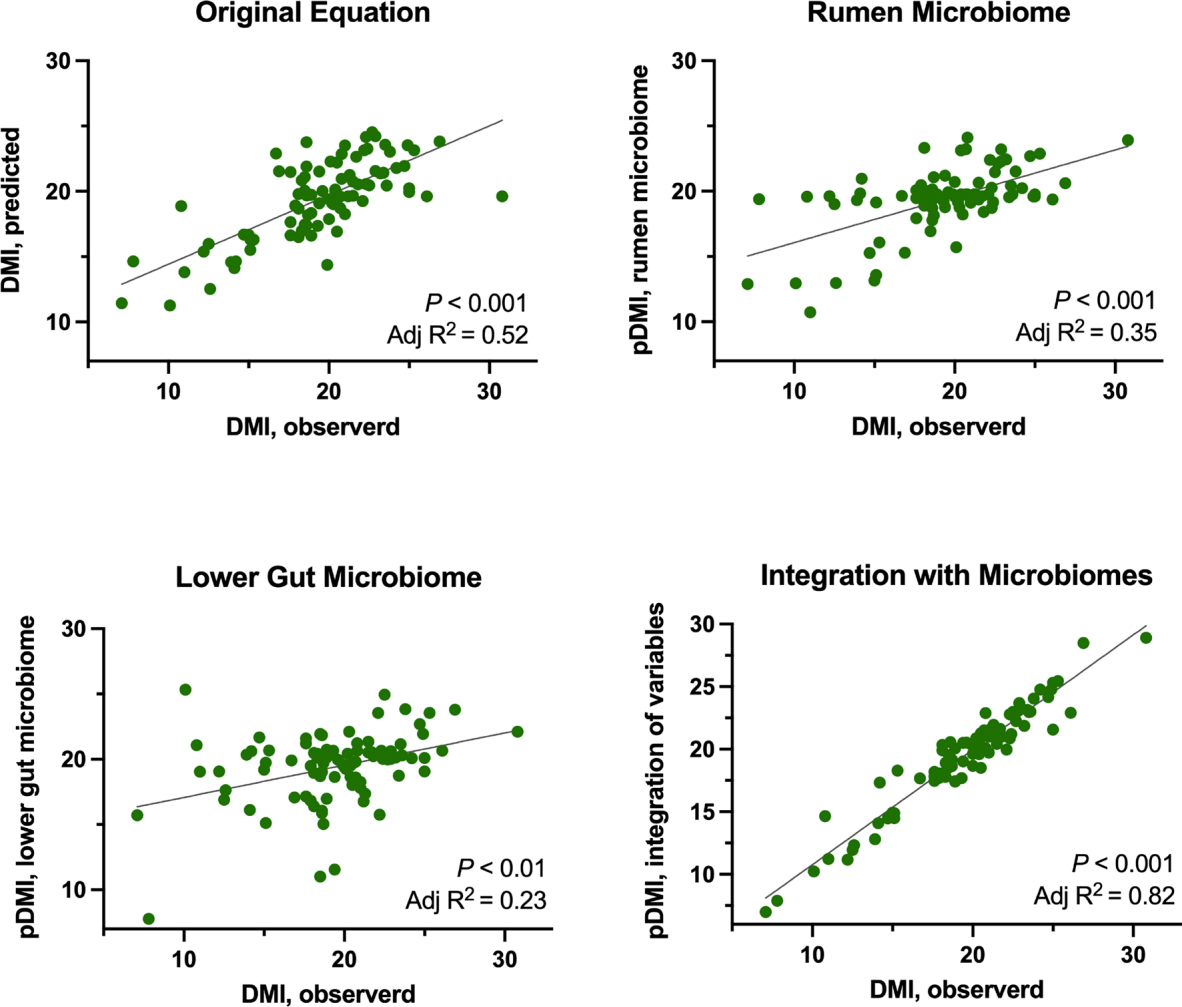
Correlation of dry matter intake with the rumen and lower gut microbiomes as well as the overall gut microbiome of lactating dairy cows. Regression was based on the original equation used for residual feed intake (RFI), followed by equations using the ASVs associated with the variables from the RFI equation corrected by dry matter intake (DMI; kg/d) from either the rumen or lower gut microbiomes, and equations integrating the RFI variables with the ASVs associated with RFI variables corrected by DMI (kg/d) from both the rumen and lower gut microbiomes. Significance was declared at *P* ≤ 0.05.

One of the dairy industry's most significant challenges is finding alternatives that make a dairy herd more profitable by optimizing the conversion of the feed offered to the cows into high-quality milk. Therefore, the last goal of our study was to quantify the contribution of the rumen and lower gut microbiomes to the current standard calculation of feed intake and production efficiency in dairy cows. Our results show the ASVs associated with the efficiency of each RFI variable in both the rumen and lower gut microbiomes explain a significant part of the variation in DMI (kg/d; Fig. 6). The original equation used to calculate DMI was considerably improved as the rumen and lower gut microbiomes were added to the equation. The final calculated integration containing the original variables used to calculate DMI and the associated ASVs from the rumen and lower gut microbiomes accounted for a high variability in its equation (adjusted R^2^ = 0.52 to 0.82, respectively).

**Figure 7:**
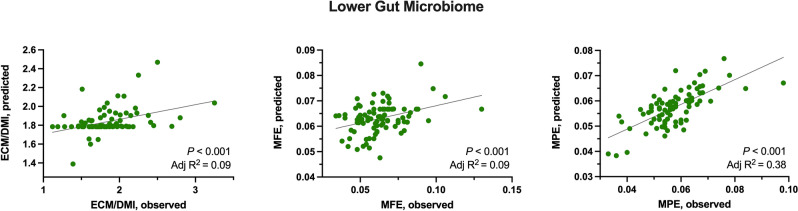
Correlation of the lower gut microbiome with each associated milk production efficiency trait (kg/kg of DMI) in the linear discriminant analysis of effect size (LEfSe). Associated traits were milk production efficiency (ECM/DMI), milk fat production efficiency (MFE), and milk protein production efficiency (MPE). Significance was declared at *P* ≤ 0.05.

Regarding the efficiency of these cows to produce ECM, milk fat, and protein, a regression using the bacteria from the lower gut microbiome associated with each variable was performed and is summarized in Fig. 7. Although the contribution of the lower gut microbiome composition to the efficiency of milk and milk fat production was low (adjusted R^2^ = 0.09 for both variables), the lower gut microbiome moderately explains the variability in milk protein production efficiency (adjusted R^2^ = 0.38). The latter correlation likely follows a similar pattern explained earlier for the ASVs associated with the efficiency traits. The microorganisms present in the lower gut give a general overview of the nutrient digestibility in previous compartments of the GI tract^20^. More efficient animals are likely to produce more short-chain fatty acids and microbial protein production in the rumen from highly fermentable carbohydrates fermentation^1,8^. Because these end-products of fermentation are directly associated with greater milk production^20,37^, the decrease of these nutrients reaching the lower gut allows the growth of microorganisms linked to more undigested nutrients; subsequently, creating the link with production efficiency.

Prediction of host-phenotypes based on the GI microbiomes has been targeted in several human disease studies^38–40^. Here, we sought to shed light on this topic in dairy cows by using the whole microbiome associated with the respective production traits to assess their contribution in the variability of feed efficiency. Previous studies investigating the ruminant gut microbiome evaluated the association of specific microorganisms with production traits^1,7,8^. In contrast, we used the whole core microbiome in the current study to understand their overall contribution to such traits. Because previous *Bos indicus* studies in beef cattle had reported an association of the lower gut microbiome with feed efficiency^10^, we investigated and observed a similar association in dairy cows. Future studies need further investigate the lower gut as a critical fermentation site contributing to ruminants' feed and production efficiency.

Overall, the rumen and lower gut microbiomes of the first lactation Holstein cows were stable over the main stages of lactation. Although we also observed associations of production traits with both microbiomes as in previous studies, these were weaker after correcting these variables for feed intake. This study shows a greater association of the lower gut microbiome of dairy cows with the efficiency of milk, milk fat, and milk protein production. These associations may be due to the nutrients reaching the lower gut of these animals and potential greater ability of these microbiomes in harvesting more of such nutrients in the lower gut. The most remarkable association found in the lower gut microbiome was milk protein production efficiency. Interestingly, integrating the core rumen and lower gut microbiomes associated with feed efficiency considerably increased the accounted variability in feed intake. This study clarifies significant drivers of production efficiency in ruminants and may guide future research towards more sustainable livestock production.

## Materials and methods

Experimental procedures were approved by the University of Illinois at Urbana-Champaign Institutional Animal Care and Use Committee (IACUC; protocol #15200). All ARRIVE and IACUC guidelines and regulations were followed during the entire duration of the study. Eighteen first lactation Holstein dairy cows from the same cohort with mean body weight (BW) of 553 ± 32 kg were enrolled in this longitudinal study right after calving. All cows were housed in the same tie-stall barn at the Dairy Unit, Department of Animal Science, University of Illinois Urbana-Champaign. Each individual allowed feedbunk separation for feed intake measurements. Cows were fed once a day, and feed was pushed forward 4–6 times a day. All animals had free access to feed and water throughout the study.

### Experimental design and sampling procedure

Rumen and fecal samples were collected on days 7, 21, 50, 90, and 130 of lactation. These days were chosen to represent the rumen and the lower gut microbiome of the cows to respectively represent parturition, end of the transition period (3 weeks before and after calving), the peak of lactation (milk volume kg/d), the peak of DMI (kg/d), and mid-to end-of lactation periods.

Feed intake, body weight, body condition score, and milk yield were measured daily throughout the study. Samples of the total mixed ration, orts, and dietary ingredients were collected daily and analyzed for dry matter and N concentration following AOAC^41,42^. Milk yield was recorded daily using an Alpro Milk point controller 780 (DeLaval, Kansas City, MO, USA) and retrieved using a standard commercially available software (DairyComp 305, Tulare, CA, USA). Milk samples were collected weekly from daily milking times (0500 and 1700 h) and analyzed for fat, lactose, and true protein by infrared analysis (AgSource, Verona, WI, USA).

An oro-ruminal sampling device was used to collect rumen content samples 3–4 h after the morning feeding time^43^. Briefly, a vacuum pump equipped with a glass container was connected to a probe of approximately 200 cm in length and 2.5 cm in diameter before being used. The probe was inserted orally and then passed through the esophagus to reach the cow's rumen. Rumen content was collected through building vacuum pressure in the probe. The first 100 mL were discarded to avoid contamination of rumen contents with esophageal components, such as saliva and mucus. Then, 500 mL of rumen content were collected, 50 mL of the content was immediately frozen in liquid nitrogen for further analysis. The pH was measured with a portable pH meter in the remaining content. Fecal samples were collected directly from the rectum using a gloved hand at the same time points of rumen sampling to characterize the lower gut microbiome profile. Immediately after collection, ~ 100 g of feces was frozen for further analysis. Both rumen and fecal samples were stored in a − 80 °C freezer until microbial isolation, DNA extraction, and sequencing were performed.

### DNA extraction, library preparation, and sequencing

Extraction of microbial genomic DNA was done similarly to that previously reported^15^. Briefly, bacteria were isolated from the rumen and fecal samples by thawing the samples at 4 °C and subsequently centrifuging them for 10 min at 16,000 RCF in a DNase-free microcentrifuge tube. The supernatant was discarded, and the pellet resuspended in nuclease-free water. A QIAamp Stool DNA Extraction Mini Kit (Qiagen) was used for genomic DNA isolation. Manufacturers' instructions were followed for genomic DNA isolation after adding 400 mg of lysozyme during microbial resuspension and incubation for 12 h at 56 °C. A NanoDrop ND-1000 spectrophotometer (NanoDrop Technologies, Rockland, DE, USA) was used to determine DNA concentration and purity measurements with ratios of A_260_ to A_280_ and A_260_ to A_230_. Only samples with ratios > 1.5 were used in further analysis (10.13140/RG.2.1.2961.4807; 10.1038/s41538-019-0056-6; 10.1101/2020.05.18.102574).

Library preparation and sequencing were performed similarly to those described by^44^. Amplification was performed through polymerase chain reaction (PCR) in a Bio-Rad C1000 TouchTM Thermal Cycler (BIO-RAD, Hercules, CA, USA). The V4 region of the 16S rDNA gene was amplified using single- and dual-index (forward and reverse) primers through an initial 95 °C denaturation for 5 min, followed by 30 cycles of 30 s at 95 °C, 30 s at 55 °C, 1 min at 72 °C, and 5 min for final elongation at 72 °C. The forward and reverse primers used were GTGYCAGCMGCCGCGGTAA and GGACTACNVGGGTWTCTAAT, respectively. Primers and small DNA fragments were removed using a 1% low melting agarose gel extraction kit (National Diagnostics, Atlanta, GA, USA). Purification and normalization of amplicons were performed using a SequalPrep plate kit (Invitrogen, USA), and the DNA concentration was measured with a Qubit^®^ Fluorometer. A DNA library was prepared by equally pooling all of the amplicons together; qualitative real-time PCR was used for quality check. A total of 180 samples (90 rumen and 90 fecal samples) were sequenced at the J. Roy Carver Biotechnology Center at the University of Illinois Urbana-Champaign using an Illumina MiSeq 2500. Sequences were deposited in the Sequence Read Archive (SRA) at NCBI under the BioProject accession number PRJNA777921.

### Bioinformatics and statistical analyses

The first step in our bioinformatic analyses was the preparation of our metadata. For that, average milk yields were used to calculate the concentration of milk fat, milk lactose, and milk protein. Energy corrected milk (kg/d) was calculated as previously published^45^. Residual feed intake was calculated according to Nehme Marinho et al.^46^, with the average of each day of lactation used in the study as time points. Briefly, a linear model including the fixed effects of NESec (Mcal/d), MBW (BW^0.75^), and BEC (Mcal/d) were used in the MIXED procedure of SAS 9.4. Because all cows were the first lactation, the lactation order was not included in the statistical model. The efficiency of milk production and milk components was calculated by dividing the production yield by the cow's respective DMI (kg/d). Then, cows were ranked within each variable and split into lower and higher-end consumption levels, production, or efficiency. For residual feed intake and efficiency variables, animals were grouped into least or most efficient ones. Residual feed intake variables were also corrected for DMI (kg/d) to assess ASVs associated with the efficiency of each variable. Finally, community analysis was performed by testing differences between microbiome as associated with each variable.

Denoising and community analyses of the sequenced amplicons were primarily performed in R Studio. Sequences were denoised using the *dada2* pipeline^47^, in which demultiplexed fastq files from forward and reverse readings were inspected, filtered, and trimmed based on their quality scores and error rates. Paired-end readings were merged, chimeras were removed, and ASV tables were created. Taxonomy was assigned using the 16S rRNA SILVA v138 database^48^ with the *phyloseq* package^49^. Total ASVs were then split into taxonomy levels, and the relative abundance of the ASVs within each taxonomy level was calculated using the *phyloseq* package.

Alpha-diversity indexes (Shannon, Chao 1, and Inverse Simpson) were calculated using the *microbiome* and *vegan* packages^50,51^. For the remaining analyses, instead of choosing a specific cutoff for the number of ASVs to be used (e.g., 100 most abundant ASVs or ASVs more abundant than a given percentage), we precisely selected relevant ASVs for the analyses using a pipeline called prevalence interval for microbiome evaluation (PIME; *pime* package)^52^. With PIME, low abundance ASVs relevant to a given group in the analysis are not discarded. Random forest classification is applied in the dataset to determine an appropriate prevalence interval for each variable of interest. Amplicon sequence variants shared among experimental units from the same group at a determined minimum prevalence are kept to visualize the differences among microbial communities.

Principal coordinate analysis (PCoA) was performed using the *pime* package, and graphs were designed using the *ggplot2*, *dplyr*, *hrbrthemes*, *viridis*, *ggsci*, and *RColorBrewer* packages. Similarly, a permutational multivariate analysis of variance (PERMANOVA)^53^ was performed for each variable to test their microbial community's dispersion differences. Except for the variable day, a model containing the effect of day, the interaction between day and the variable of interest, and the variable of interest itself were used for PERMANOVA. Linear discriminant analysis of effect size (LEfSe)^54^ was used to evaluate ASVs differences to associate with the variables in the metadata. Subclasses were used for multiple testing corrections. The data were displayed using LDA score plots for significant ASVs after Kruskal–Wallis and Wilcoxon tests. Given the goal of discovering ASVs that could potentially be associated with production phenotypes, all ASVs displayed in the LDA plots were considered for the predictions of production phenotypes.

Finally, the organisms associated with DMI and each RFI parameter corrected for DMI in the rumen and lower gut were used in linear regression to understand how much of the variation in DMI (kg/d) could be explained by the microbiome. The calculation of DMI were performed using the original equation for RFI calculation^46^, one for each of the microbiomes, and the last one when all the parameters from the original equation and ASVs from both sites of the GI tract were taken into account. The lower gut microbiome associated with each efficiency variable in the metadata were also used to understand how much of the variation of these variables could be explained by their associated ASVs. The REG procedure in SAS 9.4 was used for model selection. Model selection was based on the lowest AICC and adjusted-R^2^. Throughout all statistical analyses, significance was declared when *P* ≤ 0.05.

## Supplementary Information

Supplementary Figure 1.

Supplementary Table 1.

## Data Availability

Sequences were deposited in the SRA at NCBI under BioProject accession https://www.ncbi.nlm.nih.gov/bioproject/PRJNA777921/.
