# Supplementary figures and images for: Rumen and lower gut microbiomes relationship with feed efficiency and production traits throughout the lactation of Holstein dairy cows
Source: Sci Rep. 2022 Mar 22;12:4904. doi: 10.1038/s41598-022-08761-5 (PMC8940958; doi:10.1038/s41598-022-08761-5)

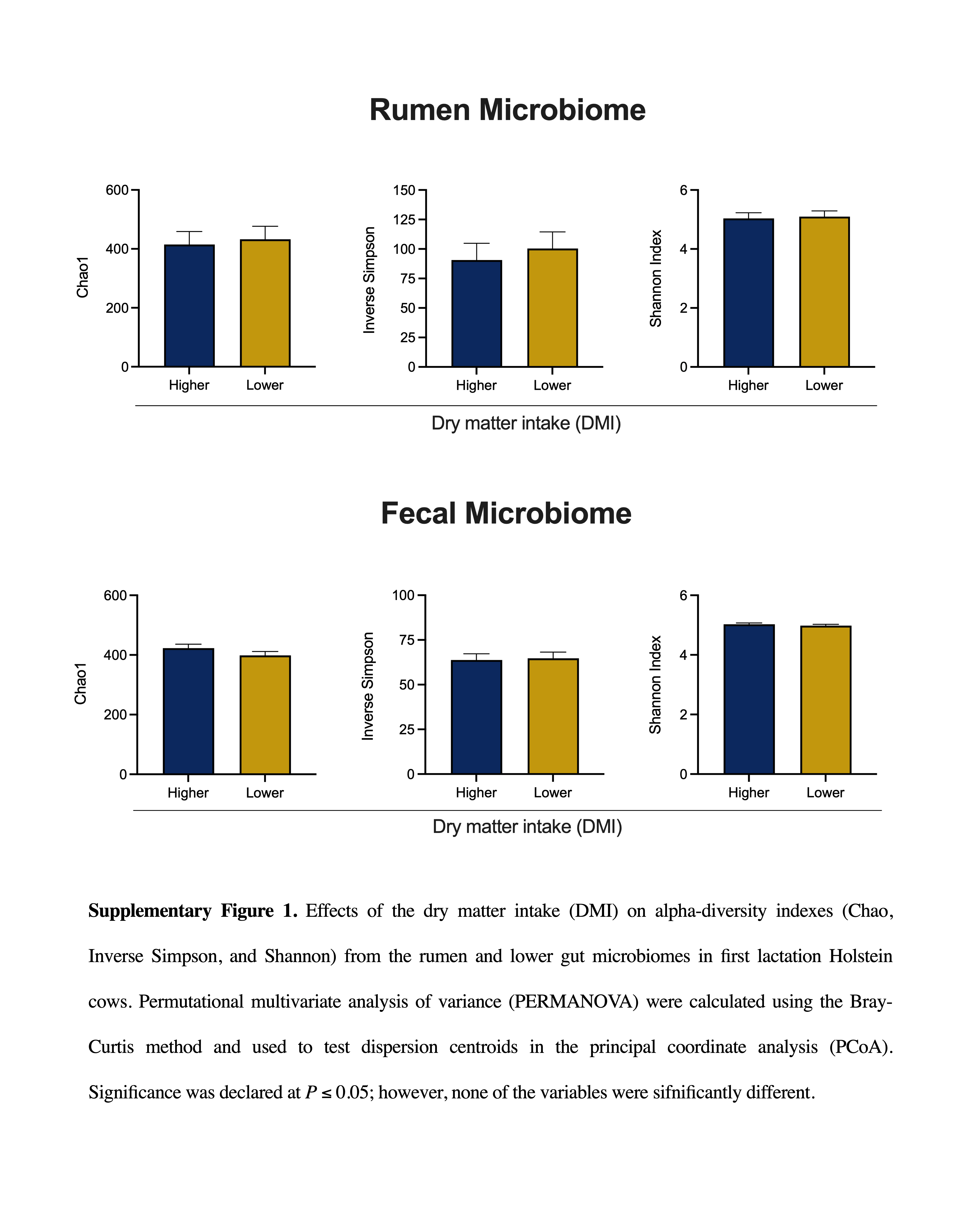

Supplement: Supplementary file 1 — Supplementary Figure 1. [file 41598_2022_8761_MOESM1_ESM.tiff]
